# Development of a maturity model for demand and capacity management in healthcare

**DOI:** 10.1186/s12913-024-11456-4

**Published:** 2024-09-23

**Authors:** Karin Myrberg, Malin Wiger, Annica Björkman

**Affiliations:** 1 Centre for Research and Development (CFUG), Region Gävleborg/Uppsala University, Gävle, Sweden; 2 Faculty of Health and Occupational Studies, Department of Caring Science, University of Gävle, Gävle, Sweden; 3 Department of Management and Engineering (IEI), Linköping University, Linköping, Sweden

**Keywords:** Demand and capacity management, Maturity model, Healthcare

## Abstract

**Background:**

The aim of this paper is to develop a maturity model (MM) for demand and capacity management (DCM) processes in healthcare settings, which yields opportunities for organisations to diagnose their planning and production processes, identify gaps in their operations and evaluate improvements.

**Methods:**

Informed by existing DCM maturity frameworks, qualitative research methods were used to develop the MM, including major adaptations and additions in the healthcare context. The development phases for maturity assessment models proposed by de Bruin et al. were used as a structure for the research procedure: (1) determination of scope, (2) design of a conceptual MM, (3) adjustments and population of the MM to the specific context and (4) test of construct and validity. An embedded single-case study was conducted for the latter two - four units divided into two hospitals with specialised outpatient care introducing a structured DCM work process. Data was collected through interviews, observations, field notes and document studies. Thematic analyses were carried out using a systematic combination of deductive and inductive analyses - an abductive approach - with the MM progressing with incremental modifications.

**Results:**

We propose a five-stage MM with six categories for assessing healthcare DCM determined in relation to patient flows (vertical alignment) and organisational levels (horizontal alignment). Our application of this model to our specific case indicates its usefulness in evaluating DCM maturity. Specifically, it reveals that transitioning from service activities to a holistic focus on patient flows during the planning process is necessary to progress to more advanced stages.

**Conclusion:**

In this paper, a model for assessing healthcare DCM and for creating roadmaps for improvements towards more mature levels has been developed and tested. To refine and finalise the model, we propose further evaluations of its usefulness and validity by including more contextual differences in patient demand and supply prerequisites.

**Supplementary Information:**

The online version contains supplementary material available at 10.1186/s12913-024-11456-4.

## Introduction

A mismatch between demand and capacity leads to a low level of access, patient queues that are too long for elective procedures, and a risk of reduced patient safety (e.g. [1] and [2–6]). In addition, demand and capacity challenges have been a recurring theme for healthcare management in recent decades (e.g. [7] and [8]). The most critical challenges on the demand side are the aging population and the consequent increase in multi-morbidity, along with the number of patients with chronic diseases requiring long-term treatment [9, 10]. In terms of capacity, the challenges include factors such as staff availability, hospital beds and financial pressures on health systems [8]. To avoid unnecessary waiting times and queues, healthcare systems should improve their responsiveness to demand [11–14]. However, adding more resources to the system is not only expensive but can also be an ineffective solution in that it can lead to decreased effectiveness as a result of concealment of poor working practices and requirement for more organisation [11]. True capacity shortages are actually infrequent as waiting lists remain constant and do not increase over time without stabilising, which would be the case if demand outstripped capacity [15, 16]. Understanding the mechanisms behind queuing and waiting times is therefore crucial [11, 17], as is matching demand and capacity more effectively [15]. Waiting lists and waiting times can thus be reduced by acquiring knowledge about accessibility and monitoring demand and capacity variations [16]. It has also been demonstrated that a focus on patient flows, i.e. the throughput of patients through several care units, is beneficial and can improve healthcare productivity [6].

A greater emphasis on production and capacity planning at various levels is a necessary ingredient to coordinate patient flows through a healthcare system [6, 18, 19]. It has been demonstrated that general, minor implementations of validated production planning and control practices within healthcare can result in major improvements in immature organisations [20]. There is no established definition of production planning in healthcare contexts, but rather various terms such as “capacity planning” or “capacity planning and control” are used [21]. However, the basic core components are the same, as illustrated by the following two examples: “Capacity planning concerns the balancing of the demand for capacity with the available capacity of the production system” [22]; “Capacity planning and control is the task of setting the effective capacity of the operation so that it can respond to the demands placed upon it. This usually means deciding how the operation should react to fluctuations in demand.” [21]. In this study, we use demand and capacity management (DCM) as the overall concept that covers the process from a strategic level to daily planning in order to balance demand and capacity supply alongside processes to meet patients´ needs, i.e. both vertical and horizontal alignment.

DCM increases an organisation’s resilience, i.e. the ability to predict its demand and capacity over a certain period, as well as the ability to deal with disruptions proactively instead of reactively [23], and maturity models (MM) are widely used in many domains for process assessment and improvement [24]. The complex nature of healthcare organisations might therefore require a model with the emphasis on cultural- and domain-specific areas [25]. Healthcare organisations consist of several decentralised specialist units with their own specialisation, needs and competences, concomitantly leading to general difficulties in managing their often shared patient flows [11]. The heterogeneity of healthcare output, the large number of core processes, as well as political and ethical obligations, further aggravates such comparisons. Based on these challenges and different conditions, as well as the need for healthcare organisations to diagnose their DCM processes, a need exists to identify gaps and evaluate improvements at management level in order to help optimise organisational settings [26]. A DCM MM that is contextualised for healthcare settings would fill a gap in this area.

This study seeks to develop a MM for healthcare DCM. It is designed to facilitate process innovation and change in this domain and, by extension, to facilitate strategic orientation toward patient flows and an effective reduction in waiting times.

## Study design

**Fig. 1:**
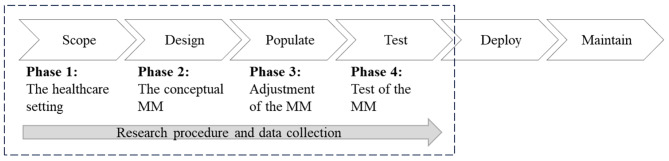
Model for development of the DCM MM modified from de Bruin et al., (2005) p. 2

Using qualitative research methods, the MM was developed through an iterative process in a natural context. Multiple units were used within joint analysis, i.e. a single-case embedded case study [27]. The substantial evidence derived from the case allows for logical generalisation and facilitates the broad application of information to other closely related cases [28]. The development phases for maturity assessment models proposed by de Bruin et al. [29] were used as a structure for the research procedure, see Fig. 1. Phase 1 comprised determination of the scope, i.e. a healthcare setting constituting specialised production of hospital care. Phase 2 included the design of a conceptual MM substantially informed by existing frameworks for production planning and control, and incorporating the needs of the intended target group, i.e. hospital managers, for production planning and strategies. Phase 3 concerned populating the model by deciding on the content and adjusting the conceptual model to the specific context. The identification of what was to be measured and how to measure it was achieved through empirical data from a healthcare department, its management, and four outpatient units introducing a structured DCM work process. Phase 4, the last step of the development of the MM in this article, tests both the construct of the model and its validity, as well as its reliability through assessing the four units. Empirical data was collected through the four phases of MM development within the scope of the case.

## The healthcare setting – phase 1

The decentralised Swedish healthcare system is divided into 21 self-governing regions. In the Mid-Sweden region, in which this study takes place, there are about 285,000 residents and 6,000 employees in the healthcare sector. The case department, which was blinded for confidentiality, is within specialised care and represented at two separate hospitals. An overview of departmental structure and scope is presented in Table 1. Based on extensive waiting-time problems, this department had volunteered for an organisational initiative, the introduction of a structured DCM work process within their outpatient care in conjunction with two other departments. Following Lillrank et al. [30], patient demand at the case department studied can be segmented in independent one-time visits (One visits), standardised processes (Elective Care), more iterative processes (Cure) and chronic patient flows (Cure). Workforce at the nurses’ units also include assistant nurses, medical secretaries, psychologists, dieticians and physical therapists that work solely with outpatient care. The physicians support both the outpatient services and the inpatient ward.

**Table 1:** Structure of the case department

One department divided in two hospitals		
	**Hospital A**	**Hospital B**
**Size**	Small	Medium-sized
**Physicians’ unit**	21	23
**Nurses’ unit**	27	37
**Wards**	Out- and inpatient (inpatients not included in the study)	Out- and inpatient (inpatients not included in the study)
**Individual patients treated annually**	3,250 (outpatients)	5,200 (outpatients)
**Annual appointments **	8,050 (outpatients)	12,900 (outpatients)

## Research procedure

**Fig. 2:**
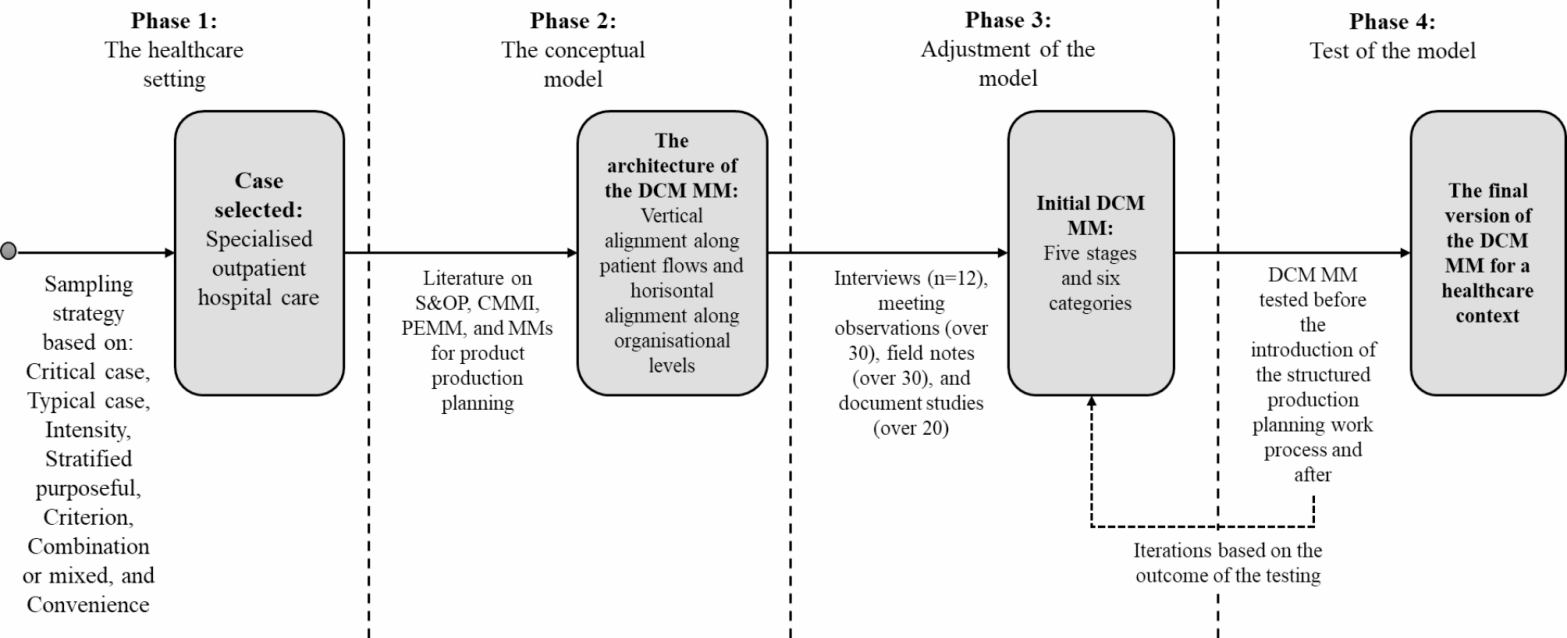
The research procedure

The primary goal of this study was to develop a production planning maturity model for a healthcare setting, which is referred to as the demand and capacity management maturity model (DCM MM) that can be used to assess the maturity level of production planning procedure in healthcare institutions. The qualitative research criteria proposed by Bryman and Bell [31] - credibility, transferability, dependability and confirmability - have been employed. The case was selected through Miles and Huberman’s [32] sampling strategies in quality inquiry, see Fig. 2 for an overview of the procedure; all data was collected from the case department and its two hospitals (phase 1).

An MM is generally a conceptual framework consisting of a set of categories and maturity stages to consider [33]. The architecture [29] of the DCM MM for the healthcare setting was informed by literature on both MMs, sales and operations planning (S&OP), and the organisation of healthcare along with the core process i.e. the patient flows (phase 2). For the adjustment (populated using Bruin et al., 2005) of the DCM MM to fit the healthcare context, data was gathered from the case units (phase 3). A total of 13 interviews were conducted on two occasions with the unit managers, in August 2022 before the introduction of the structured DCM work process, and in May 2023, 6 months after the introduction. Additionally, three employee interviews were carried out in May 2023. As the managers had testified to the low level of maturity of DCM, employee interviews were not carried out prior to the introduction, only afterwards, in May 2023, see Table 2 for details. A phenomenological approach was used in the interviews [28], with questions derived from the architecture of the conceptual DCM MM, i.e. centred on: (1) Working methods for DCM, (2) Managerial and organisational support, (3) Facilitating and hindering factors, and (4) External collaboration. The interviews were conducted by two researchers (KM and MW) online and the interview-guide was sent in advance to all participants. The semi-structured interviewing [31] enabled follow-up questions for a more in-depth understanding of statements provided such as “*Please describe what kind of organisational support you require”.* The interviews ranged from 30 to 50 min in length and were digitally recorded, transcribed verbatim and checked for accuracy. The interviewees were all women aged between 38 and 60 and all participating managers were involved in the introduction of the structured DCM work process at their unit/department during the period between the two interviews, see Table 2.

Data also included meeting observations (over 30) and field notes (over 30) centred on DCM maturity in order to add another layer of interpretation to the data collected and to provide a richer context for analysis [34]. Notes were taken by KM during one-hour organisational DCM meetings within the production support team every other week. Notes were compared with those made by another member of the production team to validate data collected. Joint reflections in relation to observations and field notes were conducted on a continuous basis throughout the research project, from data collection and analysis to the writing phase. Observations were also discussed with the members of the production support team. Likewise, documents (over 20) were analysed that contained information about the planning processes before and after the introduction of the structured DCM work process. All data was collected in a case study protocol including interview guides, transcribed interviews and field notes, and a list of documents was made to ensure data reliability [27].

**Table 2:** Overview of study participants

	Unit	Hospital	Professional experience (at study start)	Interview 1	Interview 2
First-line manager 1	Physicians’ unit	A	14 years management experience	X	X
First-line manager 2	Nurses’ unit	A	2 years management experience	X	X
First-line manager 3	Physicians’ unit	B	7 years management experience	X	X
First-line manager 4	Nurses’ unit	B	3 months management experience	X	
First-line manager 5 (acting manager)	Nurses’ unit	B	1 month management experience		X
Second-line manager 1	The department and all employees all units	A + B	4 years management experience	X	X
Employee 1	Nurses’ unit	A	15 years professional experience		X
Employee 2	Physicians’ unit	A	10 years professional experience		X
Employee 3	Nurses’ units	B	12 years professional experience		X

Interview data and observational data were analysed by means of thematic analysis [35] using a systematic combination of deductive and inductive analysis with the aim of harnessing the advantages of each, and was thus grounded in an abductive logic [36]. Transcripts and observation notes were read carefully to gain a clear understanding of the content, and text passages related to the key questions of analysis were coded and themed in the transcripts and protocols. In the first deductive step of the adjustment process (phase 3), themes were tested for transferability based on the categories of the conceptual maturity model (*meeting*,* processes*,* organisation and IT*). In the next inductive step, we examined possible new categories emerging from the empirical data beside the above themes, which yielded two additional categories added to the MM (*organisational development and mindset/culture*), along with more in-depth content for each of the model’s categories. When new observations were added to the empirical data, the deductive analytical process derived from the *adjusted* conceptual model followed by an inductive search of new patterns. The maturity stages, codes and categories were discussed in the research team and the labels and content of each of the model’s categories were adjusted throughout the process. This approach of moving back and forth between theory and data, which involves application from a theoretical framework derived from the literature and entails the generation of new themes, is described as an “iterative and reflexive process” (p. 83) [37]. The abductive analyses mitigated the gradual modification of our conceptual maturity framework, partly as a result of theoretical insights gained, but also due to the unanticipated empirical findings.

Data that did not answer the key analysis question, i.e. the development of the maturity model, was not included in the analyses. No further refinement of the MM was conducted after this step. After the model was populated, its construct and rigour was tested, an important step in MM development [29]. Researchers tested the design of our model by applying the MM to the case department using the meeting observations, field notes and interviews. Additionally, the leader, along with a member of the organisational production support team, albeit one not involved in the research project, were chosen to simultaneously test the MM. They had previously provided the researchers with input during the model adjustment phase, ensuring that the MM’s architecture was sound and relevant (face validity). Upon testing, inter-rater agreement was high and minor ambiguities were resolved through a consensus discussion. The results from the test phase are presented in a table. On completion of the analysis, quotes from the interviewees were chosen to illustrate each category for the respective unit. All quotes were translated from Swedish to English.

## The conceptual MM for DCM – phase 2

Different MMs vary immensely as they might cover entire business processes or be related to a specific area such as sales and operations planning (S&OP) processes. Several disciplines have developed and successfully adapted maturity models to evaluate the improvement of their business processes. Examples are the frequently cited Capability Maturity Model Integration (CMMI), with the original purpose of evaluating improvements for software organisations, or the Process and Enterprise Maturity Model (PEMM), primarily developed to cover business processes ( [36, 38]). It has been demonstrated that successful adaptions of generic MMs require adoption of the domain terminology and adequate descriptions of the categories [39]. Schriek et al. report that the application of generic business process MMs to healthcare processes holds several challenges due to problems addressing specific facets of the healthcare domain [40]. It is also difficult to fully compare healthcare organisations with “typical” organisations in service and manufacturing industries, characterised by loosely coupled sets of highly specialised silos with their own incentive mechanisms [41]. A rationale for developing a healthcare-specific MM was thus to provide guidance on *how and what* to develop and improve, not only diagnosing the DCM processes (as previously described by Röglinger et al., [42]).

### Production planning in healthcare

DCM plays a vital role in adjusting resource utilisation, meeting patient needs and ensuring high-quality healthcare service delivery [18]. It can be inspired by S&OP, a tool that integrates different business plans into one set of plans, thereby improving integration and communication between businesses’ functions [43]. Its main purpose is to balance supply and demand and to align the business or strategic plan with the operational plans of the firm. S&OP addresses the issue of alignment from both vertical and horizontal perspectives [44]. Vertical alignment can be referred to as ‘‘the configuration of strategies, objectives, action plans and decisions throughout the various levels of the organisation’’, while horizontal alignment can be defined in terms of ‘‘cross-functional and intrafunctional integration’’ [45]. A DCM MM for healthcare settings will thus include *levels of organisation* (vertical alignment) and *patient flows* (horizontal alignment).

### Levels of organisation – vertical alignment

The vertical alignment can typically be described using three levels: strategic, tactical and operational [46], and a further two levels that can be added in healthcare: political (for care systems that are politically controlled) from the top, and daily level at the bottom [47]. The hierarchical relationship between different levels of planning emphasises how the strategic decisions at the upper-level influence, guide and provide the framework within which production planning decisions are made for the more operational planning at the lower level, as well as follow-up from the level below to the one above [19]. The planning horizon therefore differs from the political, measured in years, to the daily, measured in days and hours [47, 18, 46]. At the strategic level, hospital management decides on the range of services offered as well as hospital volumes and capacity requirements for some years ahead [18]. For the tactical level, decisions are often made by department managers regarding estimation of demand for products or services and delivery plans [47, 46] an important role in early warning of supply and demand imbalance [44]. On a more operational level, DCM in healthcare involves decisions related to the allocation of key resources available to serve a certain demand, such as quantity of available facilities (e.g. number of beds, examination rooms, outpatient clinics), workforce availability (physicians, nurses and other professionals), equipment availability (e.g. diagnostic imaging, X-rays) and other supplies and support services [48].

### Patient flows – horizontal alignment

As with many organisations, the main flow in healthcare bisects functions for processing, and when patients pass through several care units, managing waiting times and patient queues becomes a patient flow issue, not something a single care unit can overcome ( [6, 11, 15, 16]). The need for care arises when patients seek medical attention, or even earlier, when they experience symptoms of illness, resulting in an influx of patients, creating a demand for patient appointments [41]. As patients progress through their care journey, they require various types of interventions, some of which can be anticipated, while others are not initially known to the organisation [30]. The episode of care covers the period from first contact to last contact with healthcare, as patients pass through various care functions, units, organisations and health facilities [49]. The span is wide-ranging, from telephone consulting to life-threatening conditions, and from one-time visits to a lifelong care requirement [50]. When patient cases are dealt with by several care providers, each provider manages their own care module [20], labelled as a service episode [51]. Healthcare activities can be divided into two parts, where one involves contact with the patient and the other does not [52]. The patient/provider meeting requires synchronisation [53], which is considered a service event [51]. An additional lower level of aggregation is service activities. Such activities can occur in interaction with the patient, taking X-rays for example, or merely as administrative activities related to a specific patient’s treatment, e.g. surgery scheduling or referral reviews [51]. Patient flow resources can be shared among many units or departments in all levels of aggregation and are therefore especially important in facilitating a smooth patient flow [18]. Patient flow can consequently be highly complex due to numerous interdependent parts that contribute to resolving the patients’ health problems.

### A DCM model

**Fig. 3:**
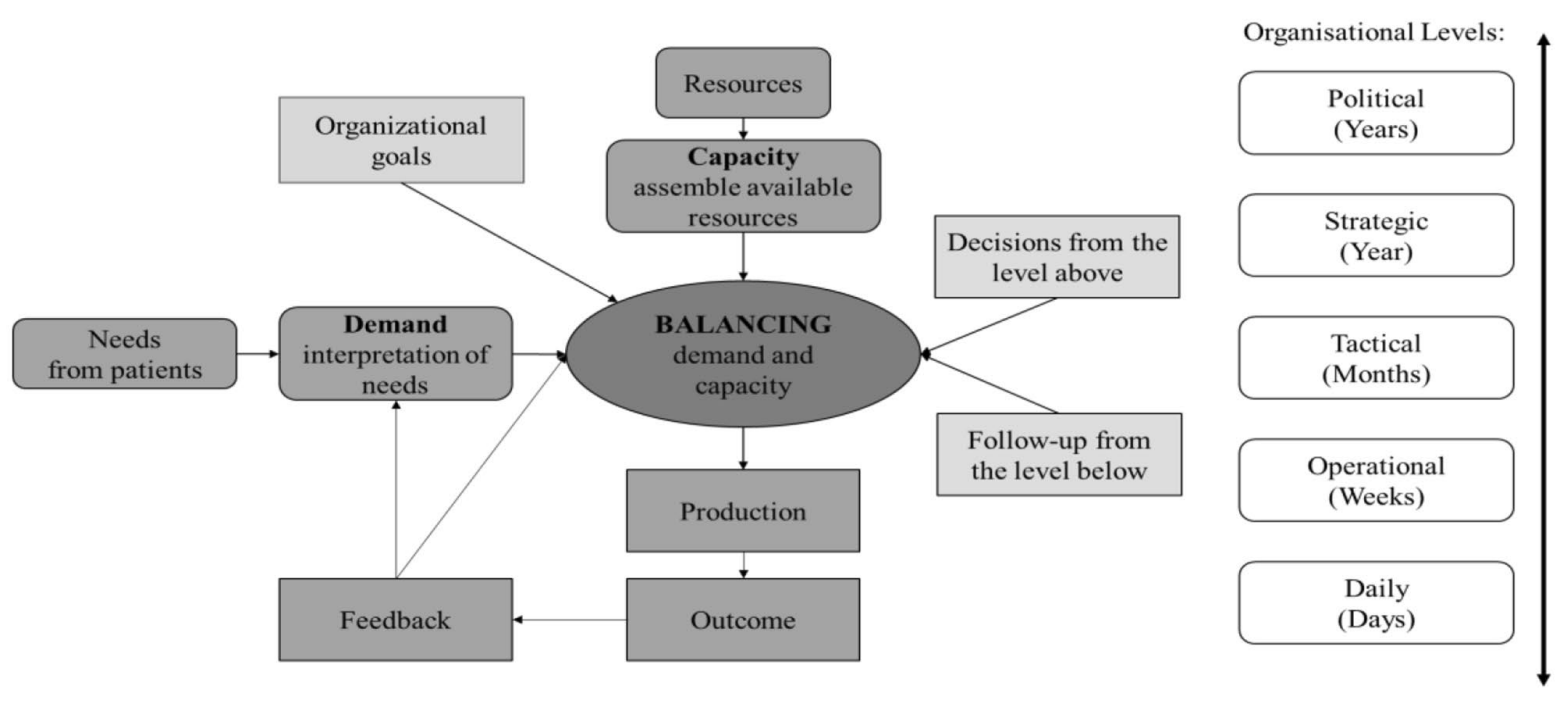
A demand and capacity management model for healthcare setting

DCM estimates the demand for healthcare services, predicts and analyses future patient needs, and forecasts resource requirements to balance demand and capacity supply and to ensure the plans and performance to support organisational goals [19]. Predicting, planning and executing activities, followed by reflecting on the outcomes and learning from them, are crucial steps in managing disruptions and building flexibility [12, 23]. This results in a feasible production plan and provision of information to the lower level of the organisation as well as follow-up feedback to both the upper level and the next planning round [19]. See Fig. 3 for a general theoretical illustration of DCM in a healthcare context.

### The architecture for the DCM MM

A frequently quoted MM for production planning is that of Grimson and Pyke [54], where they develop a comprehensive framework for production planning integration based on five key categories and propose five stages of maturity. This model was subsequently elaborated by Wagner et al. [55]. Grimson & Pyke were in all likelihood inspired by an earlier, more elementary, framework by Lapide [56], which was used for tactical planning in a healthcare context by Larsson and Fredriksson [19].

**Fig. 4:**
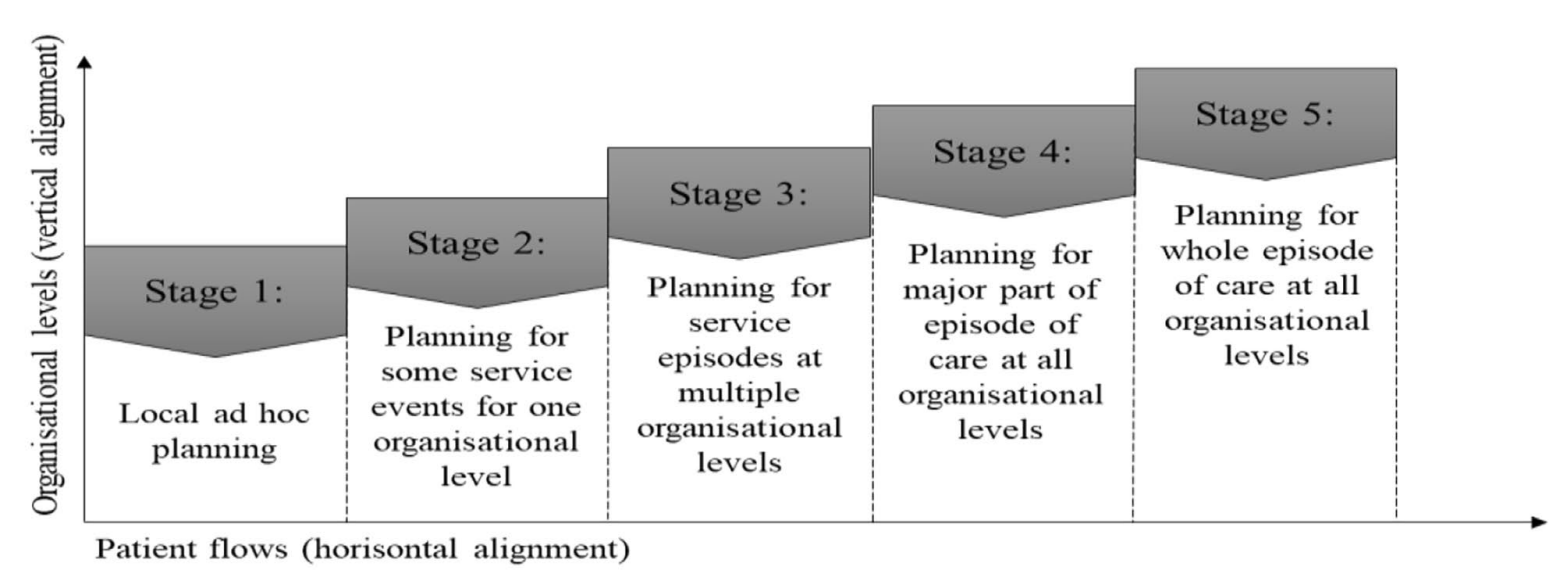
The architecture of the DCM maturity model developed in this study

These two frameworks by Lapide [56] and Grimson & Pyke [54] acted as an inspirational starting point in formulating the conceptual DCM MM. A five-stage maturity model, as pioneered by Grimson & Pyke, was considered necessary at an early stage due to the immature state of the DCM area within healthcare settings. Naturally, apart from the maturity stages, a maturity model needs a set of categories to assess [33]. For a model with a high level of specificity, though with the prerequisite that it is also easy to survey, including both organisational levels (vertical alignment) and patient flows (horizontal alignment), see Fig. 4.

## MM adjustment – phase 3

In this phase, we endeavoured to achieve a well-constructed model with relevant maturity stages and categories adjusted in relation to the empirical findings from the case, see Table 3. The most immature stage covering a low maturity extreme, originally worded “No S&OP-processes” by Grimson & Pyke, was renamed “absent”. The second maturity stage was labelled “marginal”, following the Lapide model, a stage which comprises less formal and sporadic planning. The third, more advanced stage, which includes basic elements of an S&OP process, was labelled “standard” in conformity with Grimson & Pyke, with the fourth stage labelled “class”, inspired by Lapide. In the most advanced stage, labelled “proactive” as in Grimson & Pyke, the organisation fully employs all the processes of stage 4, including a proactive way of addressing issues and a perspective on S&OP processes that permeates the whole organisation [54].

Prior to data collection, our conceptual maturity model included three of the categories from Grimson & Pyke (*meetings*,* organisation and IT*) and one (*processes*) from Lapide. During the iterative adjustment process, the organisational category from the Grimson & Pyke model was renamed “Management support” due to the large size and complexity of most healthcare organisations. Management support is a strong enabler of well-functioning S&OP processes. However, healthcare managers receive little support with tools and processes [54].

Processes is a central part of the maturity model and refers to how well the organisation constructs its demand and capacity plans and how well these plans interface. There are contextual differences between hospital departments and between hospitals within the same organisation, managers are thus allowed to operate differently. Healthcare-specific information about patient flows was added to “processes”, a category that replaced the *measurements* and *S&OP Plan integration* categories from Grimson & Pyke.

The model was supplemented with “organisational development”, as management and planning of operations reveals and provides opportunities for improvements both internally within the department as well as between care units. Lapide [57] states that it is necessary to apply a DCM culture across all functions and levels in order to be “best in class”. As changes within healthcare in the DCM area are associated with fundamental alterations to processes and routines, different groups might require different approaches in order to facilitate cultural change [46]. It is therefore necessary to examine the status of the mindset/culture. This was the rationale behind adding the category “mindset/culture” to our DCM MM for healthcare.

**Table 3:** DCM MM for healthcare

	Stage 1: Absent	Stage 2: Marginal	Stage 3: Standard	Stage 4: Class	Stage 5: Proactive
**Meetings**	1.1 No meetings • Work done by single person	1.2 Informal meetings • Sporadic scheduling • Silo meeting culture • No collaboration	1.3 Formal meetings • Routine schedule • Spotty attendance • Primarily on executive level	1.4 Formal meetings • Attendance and participation should be 100% • Employee engagement	1.5 Event-driven meetings • Scheduled to consider a change or discuss a supply–demand imbalance • Collaborative meeting culture
**Processes**	2.1 Non formal processes • No formal planning • Operations attempts to meet incoming orders	2.2 Disjointed processes • Separate demand plans • Capacity plans unaligned to demand plans • No consideration of patient flow	2.3 Interfaced processes • Demand plans reconciled • Capacity plans somewhat aligned with demand plans • Some consideration of patient flows	2.4 Integrated processes • Demand and capacity plans aligned • Limited external collaboration • Processes somewhat informed by patient flows	2.5 Extended processes • Demand and supply plans aligned internally and externally • External collaboration • Processes focused on patient flows
**Information technology**	3.1 No technology enablement • Individual managers keep own spreadsheets • Non consolidation of information • No technical support	3.2 Minimal technology enablement • Multiple spreadsheets • Some consolidation, but done manually	3.3 Standalone application interface • Standalone multi facility advanced planning and scheduling (APS) for both demand and capacity • Systems interfaced unilaterally • Centralized information • Some external data available	3.4 Applications integrated • Demand planning packages and capacity planning applications integrated • External information manually integrated • Some interface with administrative systems within organization	3.5 Full set of integrated technologies • Advanced demand and capacity planning workbench • External-facing collaborative software integrated to internal demand –supply planning systems • Full interface with administrative systems within organization
**Management support**	4.1 No management support • Lack of support • No demand and capacity organization	4.2 Sparse management support • Minimal support • A demand and capacity organization without authorities	4.3 Some management support at some levels • Elementary • Executive monitoring • Formal demand and capacity organization	4.4 Management support at several levels • Regular support • Executive participation • Compulsory educations	4.5 Full management support at all levels • Proactive involvement • Demand and capacity planning is understood as a tool for optimizing the whole organization
**Organisational development**	5.1 No formal connection • No activities connected to demand- and capacity planning	5.2 Ad hoc connections • Emerging activities	5.3 Documented connection • Structured and repeatable activities • Bottlenecks as targets	5.4 Integrated processes • Aligned and disciplined activities • Activities involving other parts of patient flows	5.5 Agile approaches • Optimized and proactive activities • Activities focused on patient flows
**Mindset/culture**	6.1 “It´s impossible” • Focus on internal resources • Contradictions between patient focus and demand and capacity planning	6.2 “It‘s complex” • Focus on how to optimize internal resources • Patient partly in focus	6.3 “It’s complex but can be done” • Focus on capacity • Patient in focus	6.4 “It’s possible” • Focus on demand and capacity • Parts of patient flows in focus	6.5 “We can do this” • Solution-oriented • Patient flows in focus

## Testing the MM – phase 4

In this phase, the DCM MM was tested in the case organisation, i.e. four different care units at two hospitals before and after introductions of a structured DCM work process.

The recommendations and structures for DCM at an organisational level were rather vague at the outset of the study. A recommendation for all departments was to submit a yearly production plan to a production support team. Second-line managers were offered voluntary visits from a member of the team, providing monthly opportunities to observe and discuss production figures, primarily at overall levels.

The structured DCM process led by a production support team within the organisation started by scrutinising the outpatient wards in terms of workforce ability, patient flows, booking policies and scheduling. The production support team introduced an Excel-based planning tool consisting of several coherent spreadsheets with automated data input from the regional healthcare database. Employee capacity was manually surveyed with the goal of targeting a standard week for every activity, both patient-related and other scheduled activities (meetings, assisting, educational events, training and referral-teams). The output of this specification was to monitor the time available for outpatient appointments, as well as a basis for organisational development.

Forecasts of patient demands on a rolling 12-month horizon were automatically based on historical data with the possibility of manual adjustment for anticipated trends in diseases/treatments. Patients with waiting times longer than the stipulated or medically justified upper limit were identified by the tool. Furthermore, it was possible to set a time frame within which standard patient inflow could be managed. Managers were also able to adjust seasonal influences in the tool. Time modules for different types of appointments were added to the model and it was thus possible to balance capacity available for outpatient appointments with actual demand. A worksheet displayed weekly results with a visual overview of weekly demand, actual production rate and outcome in relation to the weekly plan. This spreadsheet was central to the managers’ work at an operational level as they were able to react to deviations to the plan. Production plans for the forthcoming 6 months were updated every other month. This timeframe was chosen in order to fit the scheduling period, with the aim that the production plan would lead to adjustments to scheduling decisions. The production support team provided all managers with a comprehensive introduction to the tool along with written instructions and regular support in using it. They also had a 30-minute check-in with each manager every other week. The introduction of a structured DCM process started in September 2022 and support was gradually phased out after June 2023. One of the authors (KM) was a member of the production support team, which means that the study addresses participatory action research [28].

The test of the MM in the case department presented in Table 4, demonstrates that the managers’ use of the planning process differs. Prior to the introduction of the structured work process when there were no formal planning procedures, DCM maturity depended on the unit managers’ commitment and interest. No IT solution was available that enabled an overview of the balance between demand and capacity at the start of the study. Planning was based solely on last year’s production and did not provide a feasible plan. At consistently low levels and leaving considerable room for improvement in the DCM area, maturity levels and work processes differed between the units, even though they belonged to the same department and treated more or less the same patients.

**Table 4:** Result matrix

		Physicians’ unit Hosiptal A	Nurses’ unit Hospital A	Physicians’ unit Hospital B	Nurses’ unit Hospital B	Department management Hospital A + B
**Meetings**	before	1.2 Marginal *“We don’t look into it much. Because there are always explanations for the way things look). And the pandemic we’ve been through doesn’t make things easier”*	1.2 Marginal *“We have organized a waiting list group. We go through our waiting lists and have a closer look on things that have gotten stuck”*	1.1 Absent *”I think we should at least compare ourselves with hospital A”*	1.1 Absent *“I think that XX (the physicians’ manager) keeps track of it*,* because even though the nurses have their own clinic*,* we are dependent on the doctors”*	1.1 Absent *“In order for it to work I think it needs something from the outside*,* some support like we have through our HR or financial support. Someone who is on the team.”*
	after	1.4 Class *“Previously*,* I was left alone with the production issues. No one else looked at it and understood it. That’s one reason why this is so good*,* that we look at it together and talk about production and why it is a certain way.”*	1.4 Class *“Just recently when we had a quality revision*,* we looked at the files together and had some doubts. Then we called a medical secretary and she came and helped us”*	1.3 Standard *“I think that we need those frequent follow-ups. But it’s hard for us to find the time”.*	1.2 Marginal *“I think that this also could be a permanent thing at our staff meetings*,* For us nurses to be more involved*,* asking questions like*,* ”This is what the previous month looked like*,* any thoughts?””*	1.3 Standard *“But just the fact that we’ve had these continuous dialogues… It feels like we have made some progress and*,* above all*,* that we are more in control than we used to be”*
**Processes**	before	2.2 Marginal *”The irregular doctors’ scheduling is quite a challenge”*	2.1 Absent *“Well I don’t have a very good grip on that (DCM)]. I rely quite much on my staff”*	1.3 Standard *“Some weeks there are more doctors scheduled for clinic than we have rooms”*	2.1 Absent *“Well at the moment*,* I must admit I don’t look at the production data”*	2.1 Absent *”We do what we are used to. It varies with the first-line managers*,* and suddenly they are replaced…Production is a tricky area to tackle in the midst of all the other issues.”*
	after	2.3 Standard *“Now we have realised that we do a lot of things apart from the patient*,* important things that take time. It reduces stress to be reminded of that.”*	2.3 Standard *“As for the nurses*,* this serves as a confirmation that they’re doing a great job*,* which is fun and rewarding to show them”*	2.3 Standard *“I have all this in my head actually. But I reckon it has some benefits in practise. Eventually someone will take over that lacks this overview”*	2.2 Marginal *”We’ve got something that is not only for the doctors. There is still a lot of work to be done though”*	2.2 Marginal *“We haven’t yet discussed these issues together in the management forum. It would have been interesting to share with each other”*
**Information Technology**	before	3.1 Absent *“It is hard to keep track of things when you only have the waiting list and the appointment schedule”*	3.1 Absent *“We got four nurses that schedule the doctors’ appointments. So they have quite an insight”*	3.1 Absent *”We only look at the waiting list”*	3.1 Absent *“Right now*,* I need to familiarise myself with these things. So where can I find those numbers?”*	3.1 Absent *”People don’t think of these things*,* they have their weekly schedule and their own waiting list to worry about”.*
	after	3.3 Standard *“I’m a bit old school. I wasn’t born with a computer in my hand*,* so if I can do it*,* then most people can”*	3.3 Standard *“You are afraid that things would disappear*,* that you would accidentally delete or destroy something”*	3.3 Standard *“I think it’s an advantage to be able to show it clearly and have it organized in a neat way.”*	3.3 Standard *”I’m not that good at Excel*,* but here you can actually understand what you see”.*	3.3 Standard *“We now got a tool*,* for follow-ups above all. It feels like we’ll be able to handle it ourselves eventually”*
**Management support**	before	4.2 Marginal *“Well*,* I think we are pretty much left on our own”*	4.2 Marginal *“We might have discussed it at our monthly management forum. It’s been more a case of saying “Well*,* this wasn’t good”*	4.2 Marginal *“It’s not on the agenda. We have new managers with little insight own the matter. The previous ones*,* well they might have been partially familiar with it.”*	4.2 Marginal *“Well I’m sure it will be in focus eventually”*	4.2 Marginal *“The issue doesn’t get any focus in our management. Or maybe a little*,* but mostly in terms of economics and the like”*
	after	4.2 Marginal *“We definitely talk about production now but the responsibility is still ours. Because*,* I think it’s difficult for our managers to familiarise themselves with the everyday life here”*	4.3 Standard *“I believe there is a plan to get this to work out. It’s not just something that should be done for the sake of doing it”*	4.2 Marginal *“Well*,* I consider their support a bit vague”*	4.2 Marginal *“Production is more in focus now. So they are interested obviously*,* but in between the follow-ups*,* I don’t think there is much talk about it”*	4.2 Marginal “*I don’t’ think they have figured out how to tackle these issues. We*,* however have more structure than we had before”*
**Organizational development**	before	5.2 Marginal *“Well*,* we made a one-off effort there in the autumn”*	5.2 Marginal *“You make this effort and then you go back to business as usual and it goes on for a while*,* But after some time it’s like “oh*,* that has gotten bad*,* now we have to make an effort again””*	5.2 Marginal *“I saw that Tuesdays were always so crowded*,* and then*,* I managed to get an extra room”*	5.1 Absent *“Right now*,* it feels that no matter what you’d like to change at this place*,* the answer will be that it’s very*,* very difficult”*	5.1 Absent *“It’s not THAT bad*,* but there are certainly things to do. Right now*,* everybody digs where they stand”*
	after	5.3 Standard *“Since direct patient contact is actually quite a small part of my doctors’ total work hours*,* it’s probably redundant to tweak and change their actual total patient time”*	5.3 Standard *“I’m now able to focus on my own staff. It’s always the doctors and their production we follow. But now we’ve also got a better opportunity to improve nurses’ situation”*	5.2 Marginal *“It helps us visualise things*,* but it won’t automatically change anything”*	5.1 Absent *”If we just keep track of planning*,* adjust the schedule and appointments*,* we can target the areas with the biggest needs”*	5.2 Marginal *“And the more we talk about these issues*,* address them and involve the staff*,* the more good ideas pop up. But if we never show it*,* it won’t happen”*
**Mindset/culture**	before	6.3 Standard *”I definitively think these issues can be improved”*	6.2 Marginal *“It is so hard to grasp how many patients are waiting*,* it feels like a big cloud”*	6.1 Absent *” So it’s an extremely complex system and I find it difficult to see how it can be planned in any other way”*	6.2 Marginal *“I’m a bit worried about how I can manage to fit this in timewise”*	6.3 Standard *”And a big challenge is that we are stuck in a rut. That is something that I hope will improve”*
	After	6.3 Standard *“I think this work is fun and interesting*,* but it is not easy”*	6.3 Standard *”It makes sense*,* but at the same time it is complex*,* no doubt”*	6.2 Marginal *“Even though you can revise your plan*,* it’s still hard since many things are out of your control. There are plenty of things you can’t plan for.”*	6.3 Standard *“I think we’ll be encouraged to see that our efforts pay off*,* because that’s clearly shown here. But there’s still a lot of work to be done”*	6.4 Class *“Now we have a responsibility to keep track of what the next step is and that while we may not be able to take three steps at the time. With each step we’ll get further”*

When re-assessing DCM maturity after 6 months, it was clear that the structured work process evened out many of the differences between the units. However, the cultural resistance and mindset of the physicians’ unit in Hospital B seemed to affect the overall maturity. Scheduled meetings with a clear DCM agenda placed Hospital A at the “class” stage, whereas irregular attendance and the resignation of the nurses’ manager placed the units at Hospital B and department management at lower stages.

The general view among the testers was that the proposed MM was feasible and that it was fairly easy to carry out the assessment. The categories that left most room for subjectivity were the “processes” and “mindset/culture” categories. Here some units were initially placed in-between maturity stages.

## Discussion

The aim of this study was to develop a model to allow healthcare departments to assess DCM maturity. Using the development phases as proposed by Bruin et al. [29] as a structure for the research procedure, along with an abductive approach, enabled us to create a conceptual model that was adjusted and tested within a case department consisting of four units located at two hospitals. The test phase indicated the usefulness of the MM as it clearly visualised minor advancements from immature levels. Our principal result is thus the proposal of an MM that might be an important tool for healthcare DCM and organisational development.

It is essential that MMs reflect the complexities of the domain and its audience [29]. A discrepancy exists within healthcare DCM between the body of research that focuses on detailed and advanced methods, and the needs of the healthcare organisations. Healthcare is often characterised by silo cultures [58], with limited functional and professional boundaries between different actors such as municipal care and primary/secondary care. This focus on their own resources, demand and technology further aggravates healthcare organisations’ progression to more mature DCM stages as it might be virtually impossible to conduct a planning process focused on entire patient flows. From the patients’ perspective, the view of healthcare is as “one ecosystem” and silos are of little relevance for their care journey. An emerging discussion of communication, cooperation and system integration between different actors within the Swedish care setting is therefore promising [59]. The meaning of maturity in the context studied must be understood; what we mean by maturity within healthcare DCM is determined by patient flows (vertical alignment) and organisational levels (horizontal alignment). The proposed DCM MM thus distinguishes between, for example, Larsson and Fredriksson [22] and Visser et al. [18], since it incorporates both dimensions. This calls for a systems approach linked to a strategic orientation [51] by organising for a quicker response and flexibility in improving patient flows and planning processes [59]. Introductions of structured DCM practices within healthcare can result in improvement in immature organisations [20]. As Lapide [56] puts it (p. 16), it might not get them to the most mature stage, but it “might help them get closer, yielding substantial benefits along the way”. Our aim was to develop a DCM MM for a healthcare context, yielding opportunities for organisations to diagnose their processes, identify gaps and evaluate improvements, even at very immature stages.

The practical contribution was important throughout the development of the MM. A grid model with maturity stages presented on the horizontal axis and the different categories specified on the vertical axis as presented in the Grimson & Pyke MM seemed to fulfil that purpose. One important issue was that it should be easy to survey, and also possible to use for someone who is external to the department but involved in the organisation. It has been suggested that DCM maturity should be evaluated as a whole, as the categories are aligned, however we propose that it is also possible to assess an isolated category or use the MM as facilitator for DCM discussions.

In our case, it was somewhat controversial to learn that the DCM maturity level and work processes differed so much between the units, even though they belonged to the same department and treated more or less the same patients. One reason for this might be differences in the mindset/culture category, which is closely connected with the internal motivation for change [36] which emphasises the value of adding this category to the model. Use of our proposed DCM MM for healthcare might help to balance those contextual differences as it identifies the current stages of the units. However, further testing and verification of its generalisability is needed in order to refine the model and confirm its potential.

There are some limitations which should be taken into account when interpreting the study findings. One limitation is that the applicability of the MM was only tested within the outpatients units in one department within specialised care. The case department was represented at a small- and a medium-sized hospital and results might not be automatically transferrable to other healthcare settings. In order to extend its transferability, there is consequently a need to test the MM in other settings such as primary care and inpatient care, and preferably also in other Swedish regions. Ultimately, its applicability to healthcare systems in countries outside Sweden should be assessed.

An additional limitation is that the interviews were principally conducted by a single researcher, constituting a risk for subjective perspectives and bias when posing the questions. To ensure consistency in data collection, an interview guide was used. It might also be a limitation that the first author was a full member of the production team leading the introduction process while taking the role of the researcher, since this pre-understanding of the organisation and its culture might be a hindrance to perceiving things critically [60]. However, the content of the interviews was discussed in the research group, where two of the members were not members of the organisation. Further, the case was located at one small- and one medium-sized hospital in the Swedish healthcare setting, and results might not be automatically transferrable to all types of hospitals in other countries’ healthcare systems.

This study also has some major strengths. These include the abductive research approach which created a fruitful mix between established theoretical models and new concepts derived from our empirical data. Another strength is the multidisciplinary composition of the research team, which facilitated different perspectives on the issue under investigation. Other strengths are the relatively rich amount of data in terms of interviews, meeting observations, documents and field notes, also validated by two members of the production support team. The in-depth understanding of the case provided by the insider researcher yielded more valid findings [61].

## Conclusion

This paper presents the development, including testing, of a DCM MM with its basis in existing frameworks and adjusted according to empirical data from specialised hospital care. The DCM MM matrix contains five maturity stages, from *Absent* to *Proactive*, and six categories vital for beneficial demand and capacity management, each with detailed descriptions (*Meetings*,* Processes*,* Information Technology*,* Management Support*,* Organisational Development and Mindset/Culture*). The application of the model to our case department indicates its usefulness in assessing healthcare DCM and in creating roadmaps for improvements towards more mature levels. To refine and finalise the model, we propose further evaluations of its validity. One possible way of doing so could be through the Delphi technique of using a variety of experts from the field. In order to validate the model, it could be applied to a rich variety of clinics in several healthcare organisations, preferably in longitudinal studies. To examine the usefulness and likelihood that the model will be adopted in practice, a survey for managers might be appropriate. This will finalise the last step in the progress of the MM following de Bruin et. al.’s [29] main phases of developing maturity assessment models, moving the MM from prescriptive to also being more comparative in nature.

## Electronic supplementary material

Below is the link to the electronic supplementary material.

Additional file 1: Interview Guide.

## Data Availability

No datasets were generated or analysed during the current study.
