# Supplementary material for: Development of a maturity model for demand and capacity management in healthcare
Source: BMC Health Serv Res. 2024 Sep 23;24:1109. doi: 10.1186/s12913-024-11456-4 (PMC11421199; doi:10.1186/s12913-024-11456-4)
Supplement: Supplementary file 1 — Additional file 1: Interview Guide. [file 12913_2024_11456_MOESM1_ESM.docx]

| Appendix: Interview guide |
| --- |
| *Please describe how you work with demand and capacity management (DCM) (i.e. how you work with balancing patient demand with capacity and how you plan ahead) |
| *Please describe how you keep track of patient demand (long-term, short-term and on a daily basis) |
| *Please describe your feelings/mindset in DCM-related issues? |
| *How and to what extent are your second-line managers (for employees: first-line managers and for second-line managers: healthcare direction) involved in your DCM issues? |
| *Please describe the available organizational support with DCM issues (e.g. routines, organizational support team, educations, and follow-ups). |
| *What kind of support do you wish for? |
| What are your (unit’s/department’s) main assets for successful DCM? |
| *What are your (unit’s/department’s) main challenges for successful DCM? |
| *Is there anything particular in your work processes that affects your local patient-flows (service episodes and events)? |
| *Do you coordinate your DCM processes with other departments that are involved in your patients’ episodes of care? |
| *Please describe if there are any external factors that affect your DCM? |
| *Please describe if you keep track of any Key Performance Indexes (KPIs) in your unit/department. |
|  |
